# Bottom-up synthetic biology approach for improving the efficiency of menaquinone-7 synthesis in *Bacillus subtilis*

**DOI:** 10.1186/s12934-022-01823-3

**Published:** 2022-05-28

**Authors:** Xiumin Ding, Zhiming Zheng, Genhai Zhao, Li Wang, Han Wang, Qiang Yang, Mengxue Zhang, Luyao Li, Peng Wang

**Affiliations:** 1 Institute of Intelligent Machines, Hefei Institutes of Physical Science, Chinese Academy of Sciences, Hefei , 230031 China; 2 University of Science and Technology of China, Hefei, 230026 China

**Keywords:** Menaquinone-7, *Bacillus subtilis*, Synthetic biology, Metabolic engineering, Cofactor engineering, NADH kinase

## Abstract

**Background:**

Menaquinone-7 (MK-7), which is associated with complex and tightly regulated pathways and redox imbalances, is produced at low titres in *Bacillus subtilis*. Synthetic biology provides a rational engineering principle for the transcriptional optimisation of key enzymes and the artificial creation of cofactor regeneration systems without regulatory interference. This holds great promise for alleviating pathway bottlenecks and improving the efficiency of carbon and energy utilisation.

**Results:**

We used a bottom-up synthetic biology approach for the synthetic redesign of central carbon and to improve the adaptability between material and energy metabolism in MK-7 synthesis pathways. First, the rate-limiting enzymes, 1-deoxyxylulose-5-phosphate synthase (DXS), isopentenyl-diphosphate delta-isomerase (Fni), 1-deoxyxylulose-5-phosphate reductase (DXR), isochorismate synthase (MenF), and 3-deoxy-7-phosphoheptulonate synthase (AroA) in the MK-7 pathway were sequentially overexpressed. Promoter engineering and fusion tags were used to overexpress the key enzyme MenA, and the titre of MK-7 was 39.01 mg/L. Finally, after stoichiometric calculation and optimisation of the cofactor regeneration pathway, we constructed two NADPH regeneration systems, enhanced the endogenous cofactor regeneration pathway, and introduced a heterologous NADH kinase (Pos5P) to increase the availability of NADPH for MK-7 biosynthesis. The strain expressing *pos5P* was more efficient in converting NADH to NADPH and had excellent MK-7 synthesis ability. Following three Design-Build-Test-Learn cycles, the titre of MK-7 after flask fermentation reached 53.07 mg/L, which was 4.52 times that of *B. subtilis* 168. Additionally, the artificially constructed cofactor regeneration system reduced the amount of NADH-dependent by-product lactate in the fermentation broth by 9.15%. This resulted in decreased energy loss and improved carbon conversion.

**Conclusions:**

In summary, a "high-efficiency, low-carbon, cofactor-recycling" MK-7 synthetic strain was constructed, and the strategy used in this study can be generally applied for constructing high-efficiency synthesis platforms for other terpenoids, laying the foundation for the large-scale production of high-value MK-7 as well as terpenoids.

**Supplementary Information:**

The online version contains supplementary material available at 10.1186/s12934-022-01823-3.

## Background

Menaquinone-7 (MK-7), a lipid-soluble vitamin K2, plays a vital role in all domains of life [1–3]. In bacteria, it functions as an electron carrier in the electron transport chain and is crucial for energy production [4]. Humans also require MK-7, which plays an important role in the clotting cascade, maintenance of bone metabolism, prevention of arteriosclerosis, regulation of inflammation, and neuroprotection [5, 6]. The main synthetic strains of MK-7 include *B. subtilis* 168 (BS168) which is generally recognised as a safe (GRAS) bacterial species. It has a fast growth rate and strong secretion ability. Furthermore, excellent expression systems with good genetic stability as well as abundant tools for genetic modification are available for this organism [7–9].

**Fig. 1:**
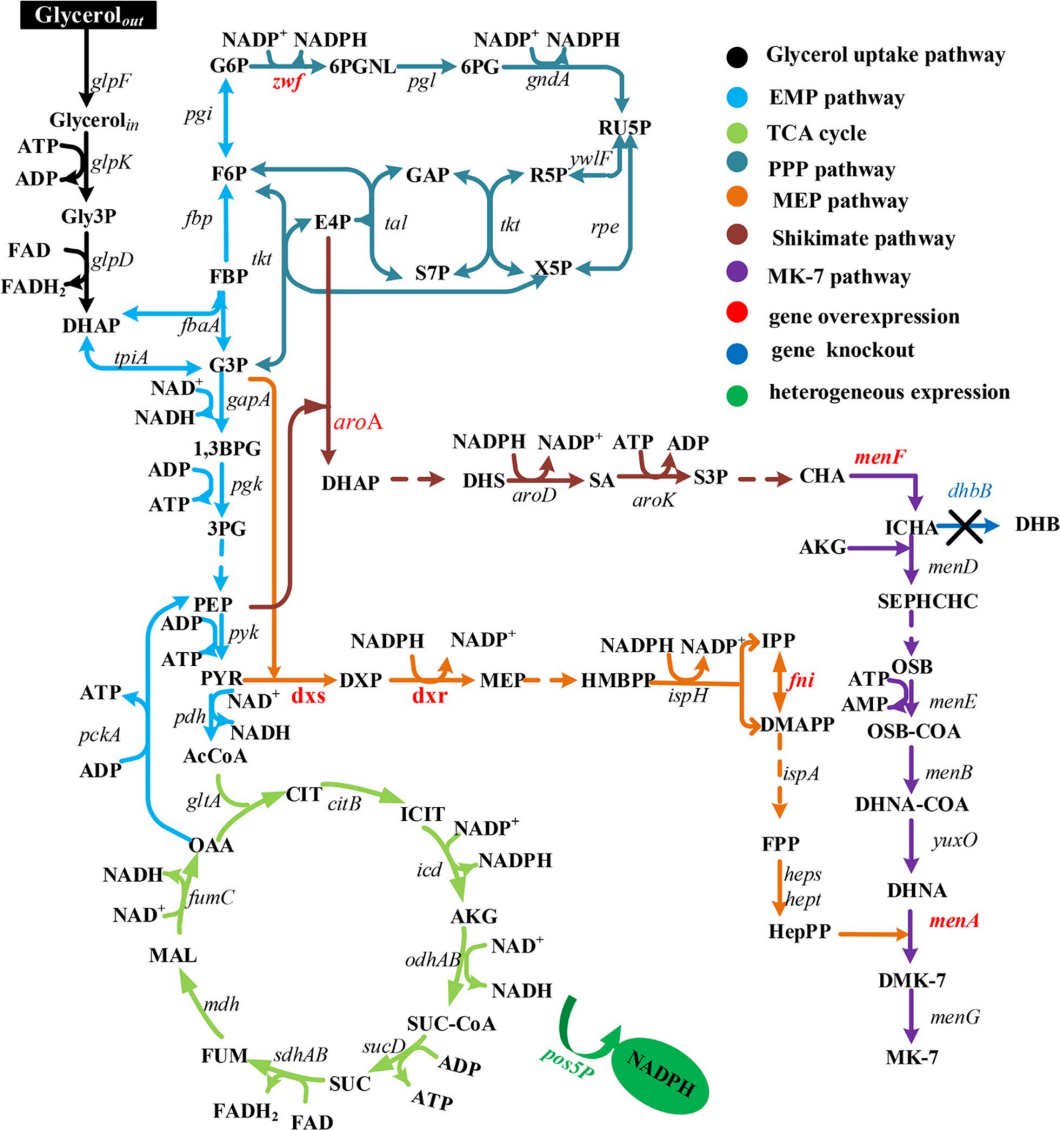
Pathways of MK-7 synthesis in *B. subtilis* 168. MK-7 biosynthesis is a complex process involving multiple metabolic pathways, such as glycerol uptake pathway, the EMP pathway, the SA pathway, the MEP pathway, the PPP pathway, the TCA cycle, and the MK-7 pathway

The MK-7 biosynthesis pathway in *B. subtilis* has been divided into seven modules, namely the glycerol uptake pathway, the MK-7 pathway, the shikimate (SA) pathway, the methylerythritol phosphate (MEP) pathway, the pentose phosphate (PPP) pathway, the glycolytic pathway (EMP), and the tricarboxylic acid (TCA) cycle (Fig. 1) [1, 10]. Thirty-seven enzymes and multiple cofactors are involved in these pathways [1]. Recently, most researchers have focused on using metabolic engineering or synthetic biology techniques to achieve a high titre, yield, and productivity (TYP) of MK-7 engineered strains. Sun et al. [11] constructed a novel synthetic menaquinone-4 (MK-4) pathway in *Pichia pastoris* via heterologous expression of *Homo sapiens* UbiA prenyltransferase containing 1 (HsUBIAD1). The production of MK-4 was increased to 0.24 mg/g dry cell weight by improving geranylgeranyl diphosphate (GGPP) supply when using menadione (VK3) as the isopentenyl acceptor. Gao et al. [12, 13]were the first to express *B. subtilis*-derived heptaprenyl diphosphate synthase component I/II (HepS/HepT) in *E. coli*. An efficient metabolically engineered *E. coli* for MK-7 synthesis was generated by combining the membrane and metabolic engineering strategies. In a subsequent shake-flask experiment, 129 mg/L of MK-7 was obtained, representing a 306-fold increase compared to the wildtype strain. Cui et al. [14] developed a bifunctional Phr60-Rap60-Spo0A quorum-sensing switch for dynamic fine-tuning of key enzymes in the synthetic pathway of MK-7 in *B. subtilis*. This led to a 40-fold improvement in MK-7 production from 9 mg/L to 360 mg/L in shake flasks and 200 mg/L in a 15-L bioreactor. Although these strategies have encouraged the MK-7 biosynthetic industry, their titres and yields are still far below market demand. The inherent constraints of the MK-7 microbial factory are related to the organism’s complex and strictly regulated metabolism, which is necessary for the coordination of different biochemical pathways and regulation of the expression and activity of key enzymes [1]. One such enzymes is 1, 4-dihydroxy-2-naphthoate heptaprenyltransferase (MenA), which is crucial for the MK-7 pathway in *Bacillus subtilis natto*. It is an intrinsic membrane protein [15]; and its abundance in natural organisms is low. However, it is difficult to overexpress this protein using genetic engineering technology because of its toxicity to host cells. The lack of techniques for inducing MenA overexpression presents an urgent problem in MK-7 synthesis. In addition, 1-deoxy-d-xylulose-5-phosphate reductoisomerase (DXR), 4-hydroxy-3-methylbut-2-enyl diphosphate reductase (IspH), and 3-phosphoshikimate 1-carboxyvinyltransferase (AroE) in the MEP and SA pathways all require NADPH as a cofactor [16, 17]. Therefore, intracellular NADPH levels may be another key factor that affects MK-7 synthesis. A study has shown that only the effective coordination of material and energy demand can significantly improve the yield of the target product [18]. When 7-dehydrocholesterol was produced in *Saccharomyces cerevisiae*, it was found that merely strengthening the acetyl-CoA module and 7-dehydrocholesterol synthesis module would result in a redox imbalance. Cofactor regeneration not only alleviates intracellular redox imbalance but also increases the production of 7-dehydrocholesterol by 75% [19].

Synthetic biology, represented by the design-build-test-earn (DBTL) cycle, provides engineering principles that allow the design and construction of biological systems with enhanced or new functions [20]. Two main approaches have been used in the field of synthetic biology. The first is the bottom-up approach. Using a single discrete genetic component to alter a module, and then extending its effects to the entire biological network, the natural function of the original biological system can be transformed or the biological system can realise a new function. This may result in "artificial life’. The second is the top-down approach that does not require deterministic understanding of genotype phenotype mapping. Instead, top-down methods expand the available genetic diversity in random (or semi-random) whole-genome engineering to create a pool of diverse variants for identifying and isolating superior phenotypes [21]. Here, we employed a bottom-up synthetic biology approach for transcriptional optimisation of key enzymes and artificially created cofactor regeneration systems to generate high-efficiency MK-7 synthesis strains. First, to shift the central carbon metabolic flow toward the MK-7 synthesis pathway, the native promoters of key enzymes and rate-limiting enzymes were replaced with either P_43_ or P_hbs_, which are both constitutive strong promoter sequences. Then, by calculating the conversion relationship between matter and energy during the synthesis of MK-7, optimising the design of the cofactor regeneration pathway, and constructing efficient NADPH regeneration systems, we increased the yield of MK-7 by 4.5 times in flask fermentation. The work described here highlights the usefulness of bottom-up synthetic biology for the development of high MK-7 titre strains and lays the foundation for optimising the fermentation and obtaining higher MK-7 yields.

## Results and discussion

### The first round of DBTL: Enhancing the metabolic flux of the MEP, SA, and MK-7 pathways

**Fig. 2:**
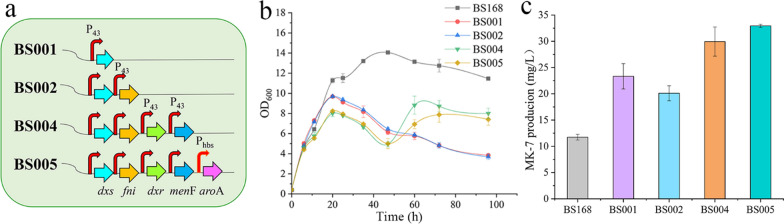
Increasing the precursor supply to push carbon into the MK-7 synthesis pathway. **a** Schematic for the overexpression of *dxs*, *fni*, *dxr*, *menF*, and *aroA* in the genome of the BS168 under the regulation of the promoters P_43_ or P_hbs_, resulting in the generation of recombinant strains BS001 through to BS005. **b** Growth curves for BS168, BS001, BS002, BS004, and BS005. **c** Production of MK-7 in BS168, BS001, BS002, BS004, and BS005 after 120 h fermentation. All experiments were performed in triplicate and error bars represent the standard deviation (SD)

The pathway for the biosynthesis of MK-7 in BS168 is shown in Fig. 1. In synthetic biology, enhancing the precursor supply and suppressing the synthesis of by-products are common strategies for improving the strain yields [14, 22]. A review of the literature related to terpenoid anabolism suggests that the reactions catalysed by DXS, Fni, and DXR are the major rate-limiting steps in the MEP pathway [7, 23]. Moreover, in the MK-7 pathway, nine enzymes are involved in the conversion of chorismate into the quinone skeleton of 1,4-dihydroxy-2-naphthoate (DHNA), and the genes encoding the first six enzymes, i.e., MenF, MenD, MenH, MenB, MenE, and MenC, are part of a gene cluster (*menFDHBEC*) in the *B. subtilis* genome [10, 22]. To improve the precursor supply for MK-7, *dxs* and *fni* were sequentially overexpressed under the control of the strong constitutive promoter P_43_, to obtain strains BS001 and BS002, respectively. Then, the native promoters of *dxr* and *menF* were replaced with P_43_ in situ to obtain strain BS004. To allow more phosphoenolpyruvate (PEP) and erythrose 4-phosphate (E4P) to flow into the SA pathway, we overexpressed *aroA* under the regulation of the P_hbs_ promoter in situ in BS004, and obtained BS005. The results of shake-flask fermentation assays showed that the titre of MK-7 from BS001 to BS005 showed an overall upward trend; however, the yield of BS002 was slightly lower than that of BS001 (Fig. 2b). Analysis of the growth curve showed that the OD_600_ of the modified recombinant strains was lower than that of the wildtype strain BS168. Among the recombinant strains, the OD_600_ decreased more significantly for the strains overexpressing *dxs* and *fni*. Therefore, owing to this poor cell growth, the production of MK-7 in BS002 was lower than that in BS001. There are several possible reasons for this observation. Some researchers believe that the overexpression of *idi* (*fni)* in the absence of the overexpression of downstream pathway genes could result in an increased flux of carbon into isoprene, thereby decreasing the carbon available for the biosynthesis of MK-7 [7]. We speculate that this may be related to the accumulation of the toxic intermediate isopentenyl pyrophosphate (IPP). George et al. [24] demonstrated that elevated IPP levels were linked to growth inhibition, reduced cell viability, and plasmid instability. In addition, endogenous *E. coli* metabolism is globally affected by IPP accumulation, a phenomenon that slows down nutrient uptake, decreases the ATP levels, and perturbs nucleotide metabolism. However, the growth performance of BS004 improved after *dxr* and *menF* were overexpressed, possibly due to the decreased accumulation of toxic intermediaries. The final yield of MK-7 in BS005 was 32.93 mg/L, which was 2.8 times that of the wildtype strain BS168. These results suggest that enhancing the supply of MK-7 precursors by increasing the transcription of rate-limiting enzymes can significantly improve MK-7 synthesis in *B. subtilis*.

### Second round of DBTL: Buttressing of the MK-7 pathway and reduction of isochorismate consumption

In the MK-7 pathway, MenA converts DHNA to membrane-bound demethylmenaquinone-7 (DMK-7), which is a key step in menaquinone biosynthesis [14, 25]. Based on the prediction of transmembrane domains (http:// www.cbs.dtu.dk/services/TMHMM/), *B. subtilis* MenA has been found to be an integral membrane protein comprising eight transmembrane helices (Additional file 1: Fig. S1). Therefore, overexpression of MenA may cause problems, such as damage-related inhibitory effects and transport system overload, in host cells [26]. Research has shown that efficient and controllable promoters are key to achieving the specific expression of target proteins. Du et al. replaced P_lacUV5_ with a rhamnose promoter (P_rhaBAD_), arabinose promoter (P_araBAD_), and tetracycline promoter (P_tet_) in BL21(DE3) to optimise the production of recombinant proteins by regulating the transcription and leakage of T7 RNAP. These engineered strains have been successfully used to improve the production of membrane proteins, including cytosine transporter protein (CodB), membrane protein foldase (YidC), and F-ATPase subunit b (Ecb) [27]. Roosild et al. discovered that Mistic, an unusual *B. subtilis* integral membrane protein, folds autonomously into the membrane—bypassing the cellular translocation machinery—and can be used as a fusion tag for the heterologous expression of eukaryotic membrane proteins in their native form in *E. coli* [28].

**Fig. 3:**
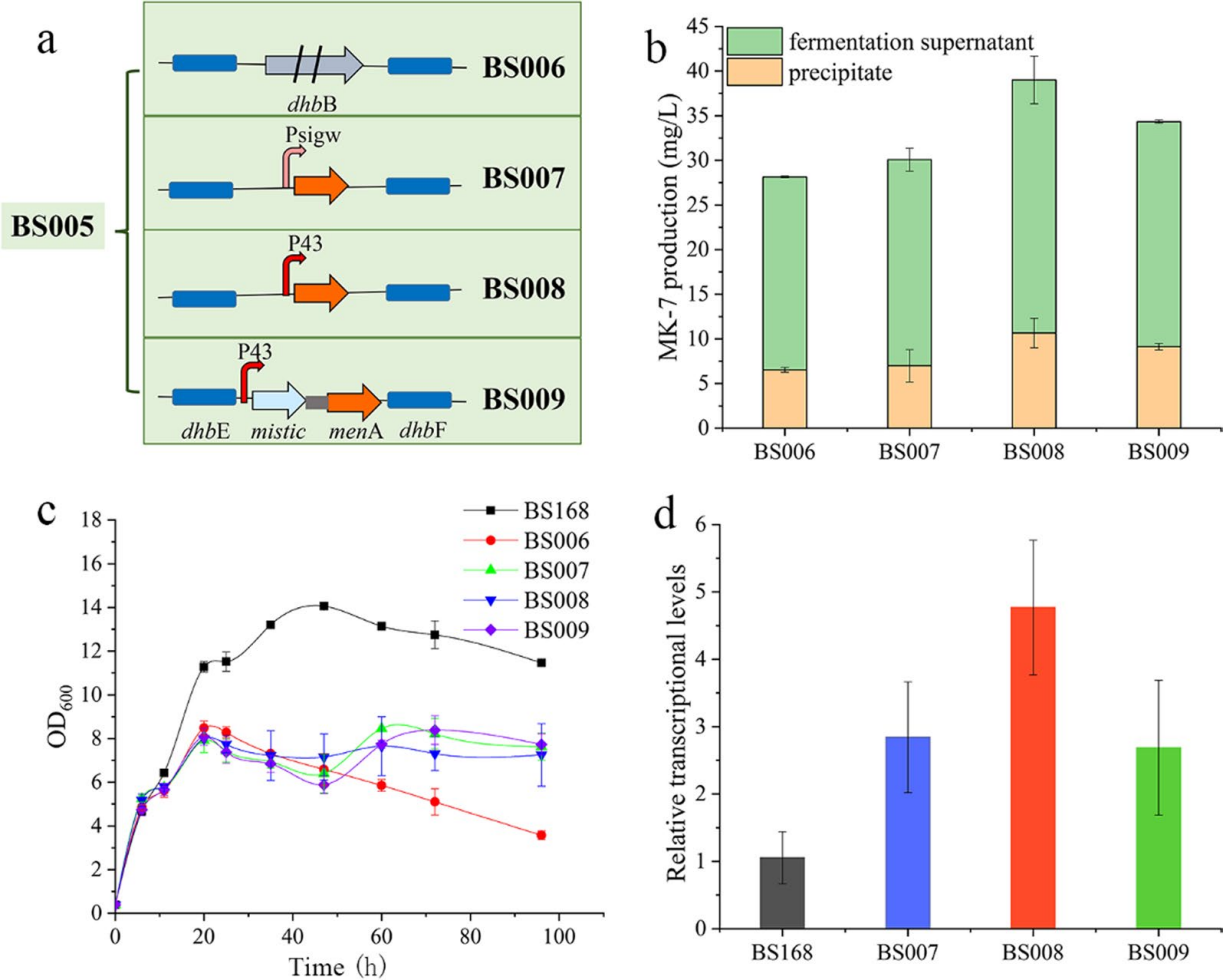
Optimising the overexpression of the key enzyme MenA and inhibiting the MK-7 pathway. **a** Schematic diagram depicting the construction of BS006, BS007, BS008, and BS009. **b** The titres of MK-7 in BS006, BS007, BS008, and BS009 after 120 h fermentation. **c** Growth curves of BS006, BS007, BS008, and BS009. **d** Relative expression of *menA* in BS168, BS007, BS008, and BS009. All experiments were performed in triplicate and error bars represent the standard deviation (SD)

With the recent expansion of the *B. subtilis* promoter database DBTBS (https://dbtbs.hgc.jp/) and the establishment of standard promoter biological element libraries, a foundation has been laid for the establishment of artificial manufacturing systems that achieve specific functions. In this study, we created the recombinant strains BS007, BS008, and BS009. To obtain BS007, we fused the medium-strength promoter P_sigw_ [29] with *menA* and integrated this construct at the *dhbB* locus of BS005. We used a similar procedure involving the constitutive strong promoter P_43_ and promoter P_43_ together with Mistic to obtain BS008 and BS009. The bacteriocin-encoding gene *dhbB* from BS005 was used to generate a negative control, i.e., BS006 (Fig. 3a). Yang et al. found that knockout of *dhbB—*responsible for the synthesis of 2,3-dihydroxy-2,3-dihydrobenzoate (DHB) from isochorismate*—*could result in the promotion of MK-7 synthesis [25]. The results of fermentation experiments showed that the titres of MK-7 in the recombinant strains BS006, BS007, BS008, and BS009 were 28.15 mg/L, 30.07 mg/L, 39.01 mg/L and 34.33 mg/L, respectively (Fig. 3b). The titre of MK-7 in BS006 was lower than that in BS005, which may have been related to the poor growth in response to *dhbB* knockout. Fortunately, the growth of BS007, BS008, and BS009 was significantly improved compared to that of BS006 (Fig. 3c). Quantitative reverse-transcriptase PCR (qRT-PCR) showed that among the three optimised expression strategies, the expression of MenA was the highest when the P_43_ promoter was used alone. The second-highest expression of MenA was achieved using the P_43_ promoter along with the fusion tag Mistic; the promoter P_sigw_ had the least effect on MenA expression (Fig. 3d). The P_sigw_ promoter is a stable phase promoter of *B. subtilis*, and therefore when the expression of *menA* is driven by this promoter, the transcription and expression of *menA* are initiated only when the bacteria enter the stable phase. This strategy not only regulates the allocation of resources between cell growth and protein production but also circumvents the disadvantages of using inducers, such as toxic side effects and leaky expression. This is a common strategy for membrane protein expression [27]. However, the expression of MenA in BS007 was the lowest, which may have been due to the short stationary period of *B. subtilis*. Additionally, the use of the strong promoter P_43_ and the fusion tag Mistic in BS009 did not significantly improve MenA expression. The specific reason is unknown, but it may be related to interference from the Mistic endogenously produced by *B. subtilis*. Our results showed that the use of the strong promoter, P_43_, to overexpress *menA* had the best effect, and in this scenario, the expression of MenA was positively correlated with MK-7 production. Overexpression of membrane proteins is a time-consuming and arduous task. In future, the number of cell membrane folds may be increased through membrane engineering strategies to accommodate more membrane proteins.

### The third round of DBTL: Improving the adaptability of material metabolism and energy metabolism

#### Calculation of MK-7 synthesis based on stoichiometry

Using the Kyoto Encylcopedia of Genes and Genomes (KEGG) and UniProt databases, we deduced the overall stoichiometry of the conversion of glycerol into MK-7 in *B. subtilis*. For calculation purposes, we assumed that all the carbon could be converted into MK-7, but the elemental balances for oxygen and hydrogen were not considered. Equations (1), (2), (3), (4), (5), (6), and (7) represent the stoichiometric formulae for the synthesis of glyceraldehyde-3-phosphate (G3P), pyruvate (PYR), PEP, E4P, DHNA, heptaprenyl diphosphate (HepPP), and DMK-7, respectively. The specific process stoichiometries are presented in Additional file 1: Note S1. Additionally, the conversion of DMK-7 into MK-7 is primarily a methylation reaction. The methyl group was provided by s-adenosyl-l-methionine [13]. The product, s-adenosyl-L-homocysteine, is first converted into L-methionine and then into L-homocysteine, which is used to regenerate s-adenosyl-L-methionine. Reaction processes that did not involve changes in NADH and NADPH were also ignored in these calculations. The reaction formulae are as follows:

1 $${\mathrm{Gly}+\mathrm{ATP}+\mathrm{FAD}}^{+} \to {\mathrm{G}3\mathrm{P}+\mathrm{ADP}+\mathrm{FADH}}_{2}$$

2 $${\mathrm{Gly}+\mathrm{NAD}}^{+}{+\mathrm{FAD}}^{+}+\mathrm{ADP} \to \mathrm{PYR}+\mathrm{NADH}+{\mathrm{FADH}}_{2}+\mathrm{ATP}$$

3 $${2\mathrm{Gly}+4\mathrm{NADP}}^{+}{+2\mathrm{FAD}}^{+}+2\mathrm{ATP} \to \mathrm{E}4\mathrm{P}+4\mathrm{NADPH}+{2\mathrm{FADH}}_{2}+2\mathrm{ADP}+{2\mathrm{CO}}_{2}\uparrow$$

4 $${2\mathrm{Gly}+{3\mathrm{NAD}}^{+}+\mathrm{NADP}}^{+}{+2\mathrm{FAD}}^{+}+3\mathrm{ADP} \to \mathrm{AKG}+\mathrm{NADPH}+3\mathrm{NADH}+{2\mathrm{FADH}}_{2}+2\mathrm{ADP}+{\mathrm{CO}}_{2}\uparrow$$

5 $${5\mathrm{Gly}+{4\mathrm{NAD}}^{+}+4\mathrm{NADP}}^{+}{+5\mathrm{FAD}}^{+}+2\mathrm{ATP} \to \mathrm{DHNA}+4\mathrm{NADPH}+4\mathrm{NADH}+{5\mathrm{FADH}}_{2}+\mathrm{ADP}+\mathrm{AMP}+{4\mathrm{CO}}_{2}\uparrow$$

6 $$14\mathrm{Gly}+{7\mathrm{NAD}}^{+}+14\mathrm{NADPH}{+14\mathrm{FAD}}^{+}+7\mathrm{CTP}+7\mathrm{ATP} \to \mathrm{HepPP}+{14\mathrm{NADP}}^{+}+7\mathrm{NADH}+{14\mathrm{FADH}}_{2}+7\mathrm{ADP}+7\mathrm{CMP}+{7\mathrm{CO}}_{2}\uparrow$$

7 $$\text{DHNA}+\text{HepPP} \to \text{DMK-}7+\text{CO}_2\uparrow$$

8 $$19\text{Gly}+11\text{NA}{{\text{D}}^{+}}+10\text{NADPH}+19\text{FA}{{\text{D}}^{+}}+7\text{CTP}+9\text{ATP} \to \text{DMK}7+10\text{NAD}{{\text{P}}^{+}}+11\text{NADH}+19\text{FAD}{{\text{H}}_{2}}+8\text{ADP}+\text{AMP}+7\text{CMP}+12\text{C}{{\text{O}}_{2}}\uparrow$$

It can be seen from Eq. (5) that 4 NADPH, 4 NADH, and 5 FADH_2_ molecules are produced when 1 mol DHNA is synthesised by *B. subtilis*, but the synthesis of HepPP is a transformation process that does not involve NADPH formation. An additional 14 mol of NADPH was required to synthesise 1 mol of HepPP (Eq. (6)). Finally, the synthesis of DMK-7 from DHNA and HepPP was catalysed by the enzyme MenA (Eq. (7)). Therefore, the total stoichiometric formula for the synthesis of DMK-7 in *B. subtilis* is (8) = (5) + (6) + (7). It was found that the synthesis of 1 mol DMK-7 requires 19 mol glycerol and 10 mol NADPH (accompanied by the production of 11 mol NADH). From the overall reaction equation, the higher the production of MK-7, the higher the level of intracellular accumulation of NADH; this conclusion was supported by the findings of a previous study [2]. However, when the intracellular reducing power is unbalanced, MK-7 synthesis is inhibited, resulting in a decrease in MK-7 levels. To rebalance the system, cells synthesise NADH-dependent by-products, which increase oxygen consumption during fermentation, reduce substrate economy, and impose pressure on industrial production. Therefore, regulating the adaptability of the material and energy metabolism during MK-7 synthesis is an urgent problem that warrants a solution.

### Rational design of data-driven cofactor regeneration pathways

Recently, with the accumulation of experimental data, the advancements in experimental technologies in systems biology and rapid development of computer technology have promoted data-driven rational biosynthesis design [30]. Numerous synthetic biology-related databases have been generated and have been used for biosynthetic analyses. e.g., KEGG [31], Rhea [32], BKM-react [33], MetaCyc [34], and Rxnfinder [30]. Based on these reaction databases, researchers have integrated metabolic reactions derived from different species to generate new artificially engineered biosynthetic pathways to obtain the desired target compounds [35]. Compared with traditional biosynthetic pathways designed based on literature searches and the professional knowledge, it is not only comprehensive, but also avoids many false trials and improves efficiency [35].

**Fig. 4:**
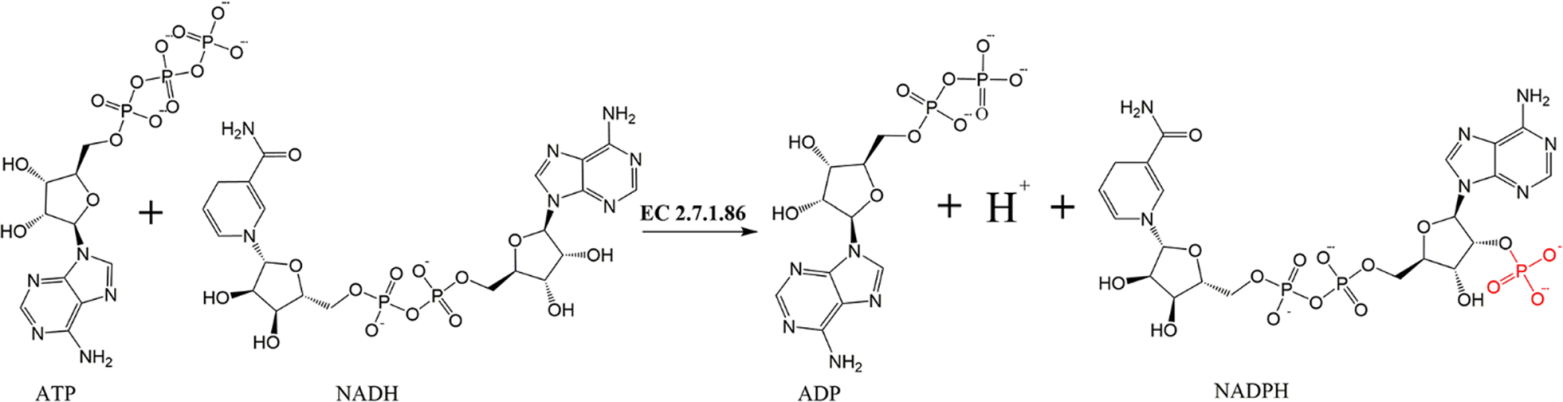
Biosynthetic pathway for the NADH kinase‒mediated conversion of NADH into NADPH

The Rhea database is a biochemical reaction resource specifically designed for annotating enzymes and genome-scale metabolic network models [35]. As *B. subtilis* does not have a native NADH-phosphorylating enzyme, we used Rhea (https://www.rhea-db.org/) to search for the NADH-to-NADPH conversion reaction pathway. The results showed that the first group of reference reactions was Rhea: 12,260, which is catalysed by NADH kinase (EC 2.7.1.86) and involves the replacement of the second hydroxyl group of ribose in NADH with a phosphate group. This reaction required ATP (Fig. 4). The second set of reference reactions was Rhea: 11,694, which involves the transfer of hydride from NADH to NADP^+^ by a transhydrogenase (EC 1.2.1.6). This reaction is driven by the movement of protons along the proton electrochemical gradient. By investigating the protein database UniprotKB and related literature, it was found that the mitochondrial Pos5P in *Saccharomyces cerevisiae* is a well-characterised NADH kinase, which has an affinity for NADH that is over 50 times higher than its affinity for NAD^+^ [36, 37]. Chen et al. constructed two types of NADPH regenerators, i.e., heterologous NADH kinase (Pos5P) and NADH kinase-like enzymes (combinations of transhydrogenase PntAB and NAD kinase YfjB) in *E. coli* to improve the availability of NADPH in S-adenosylmethionine (SAM) biosynthesis. Modulation of Pos5P resulted in superior SAM production and a yield of 5.30 mg/L SAM without L-methionine addition [36]. However, overexpression of the transhydrogenase PntAB has sometimes been associated with an undesirable reverse reaction, which involves the transfer of hydrogen from NADPH to NAD^+^ (instead of from NADH to NADP^+^). This reaction occurs because these enzymes are involved in maintaining the chemical equilibrium of the pyridine nucleotide pool [38]. After a comprehensive consideration, we intend to integrate *pos5P* from *S. cerevisiae* into the genomic metabolic network model of BS008 to improve the problem of cofactor imbalance during MK-7 synthesis.

### Regulation of endogenous cofactor systems vs creating cofactor regeneration system artificially

Cofactors can act as redox carriers to alter the intracellular redox state, adjust energy metabolism, and control carbon flux [39]. Thus, cofactor engineering is a useful strategy for enhancing the efficiency of metabolic pathways and maximising the metabolic flux toward target products [40]. Generally, increasing the NADPH supply in an organism involves reducing the NADPH consumption through competing pathways, thereby enhancing the regeneration of endogenous NADPH and conversion of NADH to NADPH [41]. This study investigated the effects of enhancing endogenous cofactor production and supplementing exogenous cofactor regeneration on MK-7 synthesis.

**Fig. 5:**
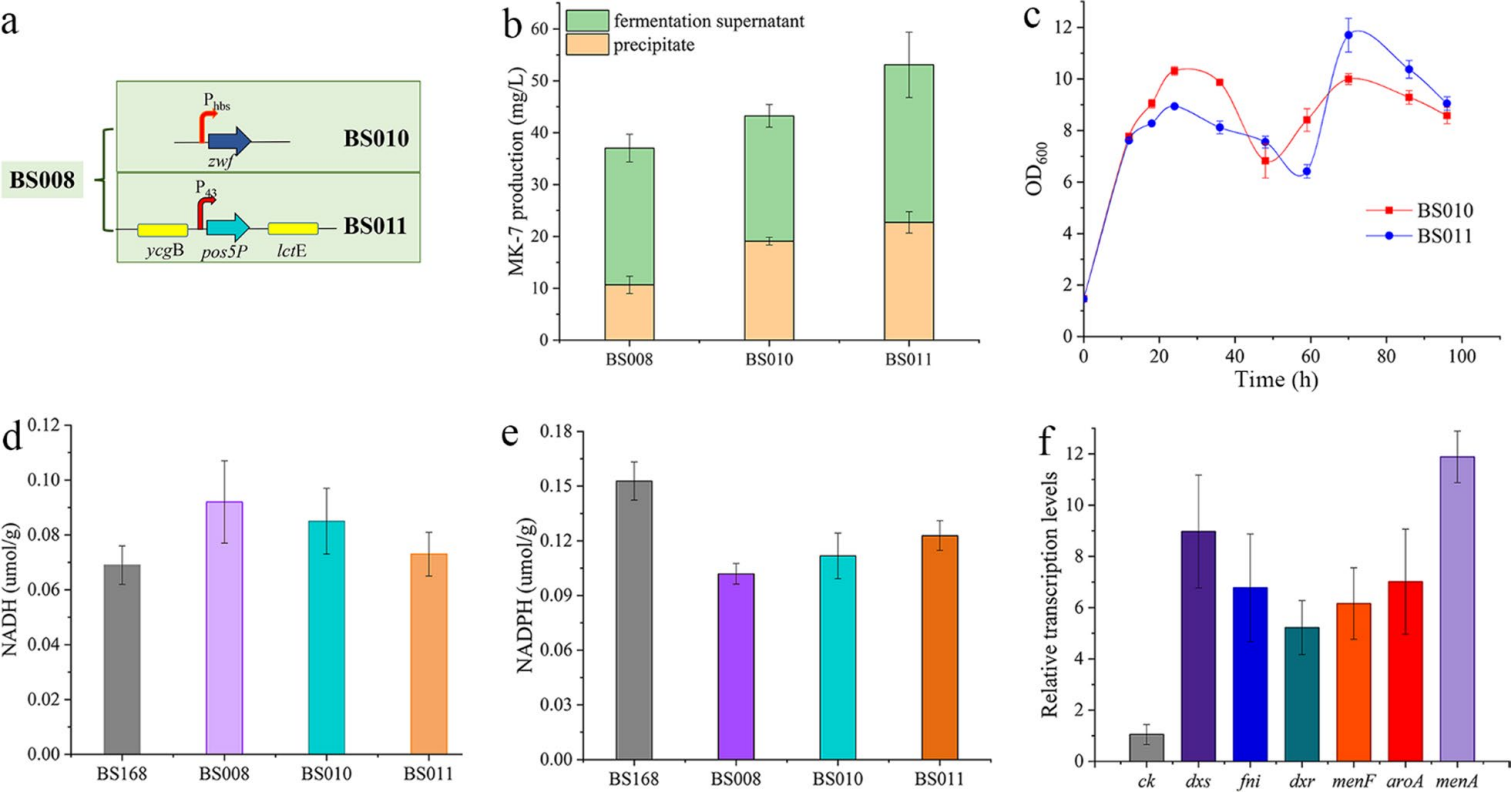
Effects of improved cofactor regeneration on MK-7 synthesis. **a** Schematic diagram depicting the construction of stains with an improved NADPH supply. **b** MK-7 production in BS008, BS010, and BS011 after 120 h fermentation. **c** Growth curves of BS010 and BS011. **d** and **e** Intracellular NADH and NADPH levels in wildtype and recombinant strains. **f** Relative expression of the modified genes in BS011 compared with that of the corresponding genes in the control strain BS168. The transcript levels of *dxs*, *dxr*, *fni*, *menF*, *aroA* and *menA* were regarded as 1 in strain BS168. Results are presented as the mean of three replicates and error bars represent the standard deviation (SD)

The PPP pathway and TCA cycle are known to be the major sources of NADPH regeneration in organisms, and glucose 6-phosphate dehydrogenase (ZWF) and 6-phosphogluconate dehydrogenase (GND) are key enzymes involved in NADPH synthesis. Studies have shown that overexpression of *zwf* and/or *gnd* can significantly increase the availability of intracellular NADPH [16, 42]. In this study, the native promoter of *zwf* in the recombinant strain BS008 was replaced with the constitutive strong promoter P_hbs_, resulting in the attainment of the strain BS010. Supplementation with exogenous cofactor regeneration mechanisms mainly involves the introduction of NADH kinase Pos5P in BS008. The 17 N-terminal amino acids of *S. cerevisiae* Pos5P are mitochondrial targeting, and exogenous expression usually requires truncation [38]. Therefore, we fused the truncated *pos5P* with the strong promoter P_43_ and integrated the construct into the *amyE* locus in the chromosome of BS008 by homologous recombination, thus resulting in the generation of the recombinant strain BS011 (Fig. 5a). Colony PCR in BS011 using primers pos5p-F and pos5p-R, resulted in a clear band at 1149 bp upon gel electrophoresis (Additional file 1: Fig. S2). The results of shake-flask fermentation showed that BS010 and BS011 could produce 43.24 mg/L and 53.07 mg/L MK-7, respectively (11% and 36% higher amounts than those in BS008) (Fig. 5b). Growth curve analysis showed that the introduction of the artificial cofactor regeneration pathway had little effect on the growth of recombinant strains, and that the growth trends of BS010 and BS011 were the same (Fig. 5c). This indicates that the artificial cofactor regeneration pathway is highly adaptable within *B. subtilis* chassis cells, which is a key factor in achieving the stabilisation and maximisation of the metabolic flux of a target pathway in an artificial cell system under unusual environmental conditions.

To further verify the effect of *zwf* and *pos5P* overexpression on NADPH levels, intracellular pyridine nucleotide concentration was measured. The results showed that the level of NADH in BS008 was 1.34 times higher than that in BS168, indicating that promoting the synthesis of MK-7 precursors resulted in the accumulation of NADH. This is consistent with the stoichiometric calculations. After the cofactor levels improved, intracellular NADPH levels of BS010 and BS011 (compared with BS008) increased by 9.7% and 20.55%, respectively, while NADH and NADH levels decreased by 7.7% and 21.15%, respectively (Fig. 5d and e). This suggests that using the NADH kinase-mediated NADPH regeneration strategy is more effective than enhancing endogenous cofactor production, which is consistent with the findings of previous reports [38]. When the effects of the two cofactor production pathways on the synthesis of MK-7 were compared, it was found that the production of MK-7 in BS010 and BS011 was significantly improved, but the effect in BS011 was even more pronounced. However, buttressing the endogenous PPP pathway to increase the NADPH level may result in the release of more CO_2_ and waste of carbon sources, which is not in line with the concept of green bio-manufacturing [36]. Therefore, strain BS011 was selected as the MK-7 synthetic strain in this study, and the relative expression of the modified genes was determined. qRT-PCR showed that the transcript levels of the overexpressed genes in BS011 were significantly higher than those in BS168. The transcript levels of *dxs* and *menA* showed the most significant changes (nine and twelve times of those observed in BS168, respectively) (Fig. 5f). In addition, the exogenous gene *pos5P* in BS011 showed higher expression (Additional file 1: Fig. S3). These results indicate that all the modified genes in BS011 were successfully overexpressed.

### Creation of a cofactor regeneration system artificially leads to the redistribution of the metabolic flux in *B. subtilis*

**Fig. 6:**
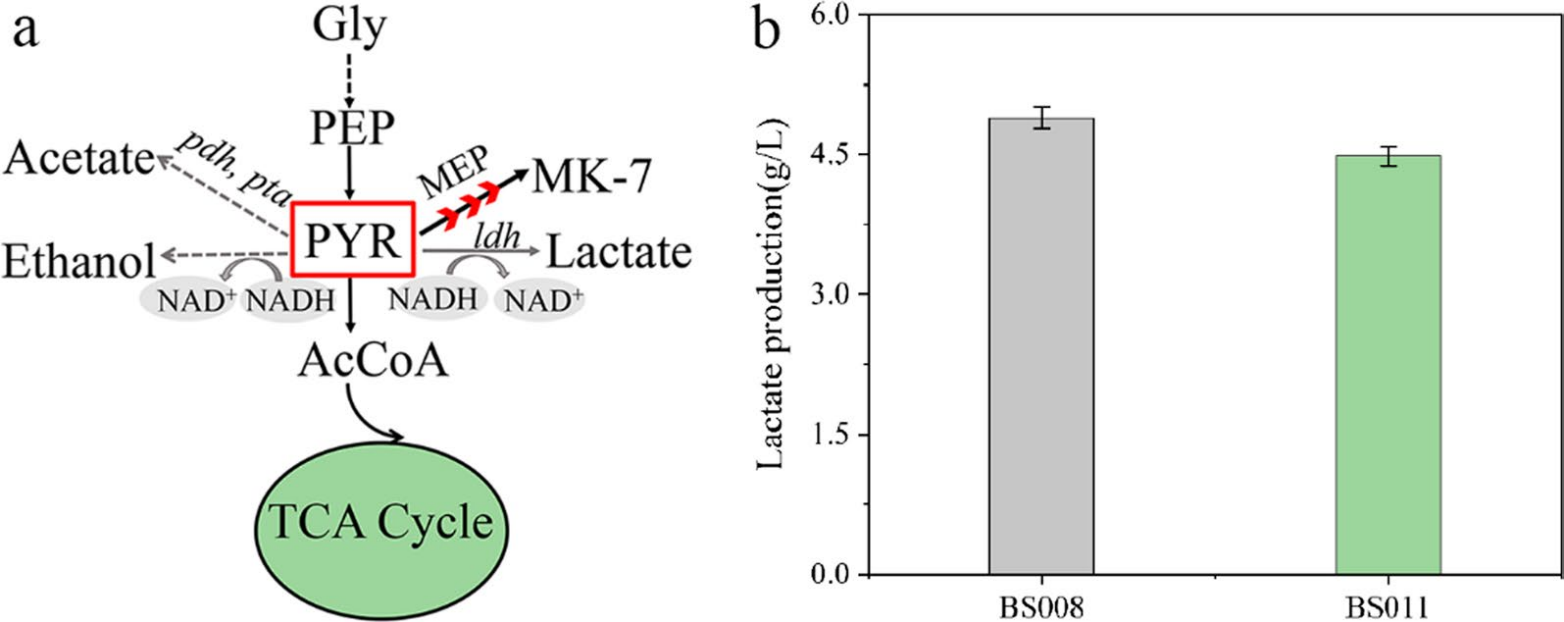
Redistribution of the metabolic flux in BS011 in response to the introduction of an NADPH regeneration system. **a** Schematic diagram depicting the central carbon metabolism flux in BS011*.* Metabolic pathways with red arrows are buttressed. **b** Lactate in the fermentation broth of BS008 and BS011 after 120 h fermentation

In *B. subtilis*, pyruvate—the final metabolite of the glycolytic pathway—is an important node in the central carbon metabolism (Fig. 6a). First, pyruvate enters the TCA cycle to generate ATP for supporting bacterial growth and metabolism. Pyruvate is a precursor of various amino acids such as alanine [43]. The entry of pyruvate and G3P into the MEP pathway is catalysed by DXS, which provides HepPP for the synthesis of MK-7. However, in conditions when the carbon metabolism is high or NADH accumulation occurs, pyruvate forms overflow metabolites, such as lactate, acetate, and ethanol. These NADH-dependent by-products consume additional carbon and reduce substrate economy [44, 45]. To characterise the redistribution of metabolic flux due to cofactor regeneration in BS011, we measured the concentrations of lactate and acetate in the fermentation broth. After testing, we found that there were no evident accumulation of acetate in either the wildtype or recombinant strains. Notably, a slight reduction in lactate formation (4.48 g/L) was observed in BS011, which was 9.15% lower than that in BS008 (Fig. 6b). This result suggests that the exchange of intracellular cofactors affects the redistribution of the carbon metabolic flux. This may be related to excess NADH being channelled to the respiratory chain to generate ATP for the conversion of NADH to NADPH, which enables the recycling of cofactors and avoids unnecessary waste of energy [36].

## Conclusions

The titre of MK-7 is generally low in most wildtype strains because of their complex and tightly regulated pathways (as well as redox imbalance). Here, we employed the bottom-up synthetic biology approach for constructing a "high-efficiency, low-carbon, cofactor-recycling" MK-7 synthetic strain. The rate-limiting enzymes DXS, Fni, DXR, MenF, and AroA, and the key enzyme, MenA, were overexpressed for transcriptional optimisation, which helped alleviate the pathway bottlenecks and promote the flow of central carbon metabolism toward the MK-7 synthesis pathway. The imbalance in reducing power during MK-7 synthesis was identified using stoichiometry. Pos5P, the key enzyme for NADPH regeneration, was screened using the Rhea database; it was then expressed in BS008 to aid the conversion of NADH into NADPH. Through three DBTL cycles, we circumvented overflow metabolism and effectively rebalanced the redox metabolism to facilitate the accumulation of the desired product, i.e., MK-7, in *B. subtilis*. A redox-balanced strain i.e., BS011—which lacked any imbalance in the context of MK-7 synthesis—was obtained with a yield of 53.07 mg/L (4.52 times that of the wildtype strain BS168). In summary, this study proposes a redox imbalance in the synthesis of MK-7, which has rarely been reported in previous studies. The unsatisfactory production of MK-7 in BS011 may be related to the low tolerance of host cells to MK-7. It has been suggested that MK-7—as a redox-active small molecule—can induce oxidative stress at high concentrations and has a toxic effect on host cells [3, 46]. Future studies will focus on improving the tolerance of chassis cells to MK-7 (unpublished data). In addition, this study mainly focused on the construction of a redox-balanced strain for producing synthetic MK-7, and the titre of MK-7 in BS011 will be further improved using fed-batch fermentation to reach high cell densities in the future. In summary, the bottom-up synthetic biology approach demonstrated here could be used as a general strategy for developing high-titre terpenoid synthesis strains with complex synthetic pathways and redox imbalances.

## Methods

### Chemicals and reagents

The organic reagents and standards used for high-performance liquid chromatography (HPLC) analysis were purchased from Sigma-Aldrich (Steinheim, Germany). All other chemicals were purchased from Sangon Biotech Co., Ltd. (Shanghai, China) and PrimeSTAR HS DNA polymerase was purchased from Takara (Dalian, China).

### Strains and culture conditions

All the strains and plasmids used in this study are listed in Additional file 1: Table S1. For the genetic manipulation experiments, all strains were cultured in Luria–Bertani (LB) broth at 37 °C and 220 rpm. Fermentation production of MK-7 was performed as follows: First, *B. subtilis* was cultured in LB broth at 37 °C and 220 rpm for 12 h, and then 10 mL seed liquid was inoculated into 500-mL flasks with 100 mL fresh fermentation medium (30 mL/L glycerol, 60 g/L soy peptone, 5 g/L yeast extract, 3 g/L K_2_HPO_4_, and 0.5 g/L MgSO_4_.7H_2_0, pH 7.3). Then, it was cultured for 120 h at 40 °C and 220 rpm. One millilitre of fermentation liquid was sampled once every 6 h, and the cell density was measured at a wavelength of 600 nm. The working concentrations of antibiotics were as follows: kanamycin (50 μg/mL), ampicillin (100 μg/mL), spectinomycin (50 μg/mL), and chloramphenicol (5 μg/mL).

### DNA Assembly and *B. subtilis* transformation

Overlap extension by polymerase chain reaction (OE-PCR) was performed as described previously [25, 47]. Both the synthesis of exogenous gene fragments and primers and sequencing of modified DNA fragments were performed by Sangon Biotech Co., Ltd. (Shanghai, China). Homologous fragments or plasmids were transformed into *B. subtilis* using Spizizen transformation [48], and the primers used for gene modification are listed in Additional file 1: Table S2.

### Gene knockout, in situ replacement of promoters, and genomic integration of heterologous genes

Most of the genetic modification strategies used in this study have been reported previously [14, 22, 47]. Gene knockout techniques mainly involve replacing the native promoters of the target gene with strong promoters P_43_ or P_hbs_. For example, the steps for obtaining the *dhbB* knockout were as follows. First, the *dhbB*-L-F/*dhbB*-L-R and *dhbB*-R-F/ *dhbB*-R-R primers were used to amplify the upstream (1000 bp) and downstream sequences (1000 bp) of *dhbB* from the *B. subtilis* genome, respectively. Then, lox71-spc-lox66 cassettes were amplified from the p7S6P43 plasmid using the spc-F/spc-R primers. These three fragments were joined using triple-fusion PCR and the purified PCR products were transformed into competent *B. subtilis* cells. Next, the spc^r^ transformants were selected. The *dhbB-knockout* strain (ΔdhbB) was obtained by two-step screening. DNA sequencing was performed using the *dhbB-L-F/dhbB-R-R* primers.

The method used for gene overexpression was similar to that used for gene knockouts. For example, the steps of *dxs* overexpression are as follows. Initially, the *dxs*-L-F/*dxs*-L-R and *dxs*-R-F/*dxs*-R-R primers were used to amplify the upstream sequences (1000 bp) and *dxs* from the *B. subtilis* genome. The *dxs*-Z-F/*dxs*-Z-R primers were used to amplify lox71-cm-lox66 and strong promoter P_43_ cassettes from plasmid p7C6P43; the remaining steps were the same as those used for the gene knockout procedure.

The heterologous gene *pos5P* was integrated at the *amyE* locus in *B. subtilis* and was expressed using the strong constitutive promoter P_43_. Four fragments, including the upstream (1000 bp) and downstream (1000 bp) sequences of *amyE*, lox71-cm-lox66, and P_43_ promoter cassettes, and the NADH kinase gene *pos5P*, were joined by triple-fusion PCR, and the purified PCR products were used to transform competent *B. subtilis* cells. *pos5P*, derived from *Saccharomyces cerevisiae* s288c, was synthesised by Sangon Biotech Co. Ltd. [45].

### MK-7 extraction

One millilitre of bacterial fermentation broth was centrifuged at 8000×*g* for 6 min, 2 mL of n-hexane and isopropanol mixture (v/v, 2:1) was added to the supernatant and the mixture was shaken; MK-7 in this mixture was extracted for 2 h. Then, 1 mL of n-butanol was added, and the mixture was shaken for 1 h. Then, the mixture was centrifuged at 9000×*g* for 5 min to separate the organic and aqueous phases; the organic layer contained MK-7. The MK-7 content in the organic phase was analysed using HPLC (Shimadzu, Japan).

The cell precipitate was extracted using a two-step ethanol method [49]. First, the cell precipitate was dissolved in 1 mL ethanol, shaken at 37 °C at 200 rpm for 30 min, and then centrifuged at 25 °C at 12,000×*g* for 10 min to collect the organic phase, after which 1 mL ethanol was used to resuspend the precipitate. After shaking for 30 min, the mixture was centrifuged at 9000×*g* for 10 min and the organic phase was collected. The organic phases were combined after the two steps and were then filtered with a 20 µm organic membrane to obtain an extract containing intracellular MK-7.

### Detection of NADH and NADPH

Intracellular NADH and NADPH concentrations were determined using a coenzyme (NAD^+^/NADH) assay kit and a coenzyme II (NADP^+^/NADPH) assay kit (Beyotime, Shanghai, China), respectively, according to the manufacturer's instructions. Cells in the logarithmic growth phase were collected, washed with ice-cold PBS, immediately frozen in liquid nitrogen, and pretreated using a homogeniser. Add the ice-cold extract and centrifuge at 4 °C, 10,000×*g* for 6 min, and the supernatants were used for the measurement of NADH and NADPH. Intracellular NADH and NADPH concentrations were quantified by a colorimetric assay at 450 nm using SpectraMax i3x (Molecular Devices, USA). Meanwhile, the protein concentration of the supernatants was measured using the Enhanced BCA Protein Assay Kit (Beyotime, Shanghai, China), and the protein content was calculated according to the empirical calibration equation (protein content = 0.5489OD_562_ + 0.0435).

### qRT-PCR

Cells were harvested during the logarithmic growth phase from the shake-flask cultures. Total RNA was purified using the RNA-easy Isolation Reagent (Vazyme, Nanjing, China). MonScript™ 5 × RTIII All-in-One Mix (Monad, Suzhou, China) was used to synthesise cDNA from total RNA, according to the manufacturer’s instructions. qRT-PCR was performed on a LightCycle480 (Roche, Germany). qRT-PCRs were performed in a total volume of 20 μL (MonAmp™Fast SYBR® Green qPCR Mix (Monad, Suzhou, China), SYBR mix, 10 μL; cDNA, 1.5 μL; final primer concentration, 200 nM). The PCR conditions were as follows: denaturation at 95 °C for 30 s, 40 two-step cycles at 95 °C for 5 s and 55 °C for 5 s. *hbs* was used as the internal control to determine relative gene expression levels. Data were analyzed using the 2 − ΔΔCt method.

### Analytical methods

HPLC detection: The production of MK-7 was determined by HPLC with a UV detector (248 nm) and C18 ODS column (Shimadzu, Japan). The mobile phase was a mixture of methanol and dichloromethane (v/v, 4:1); the flow rate was 1 mL/min, and the oven temperature was 35 °C, which was selected for calibration and analysis. Determination of glycerol, acetate, and lactate: The fermentation broth was centrifuged at 10,000×*g* at 4 °C for 5 min. Then, 1 mL of the supernatant was taken and diluted to 3 mL with ultrapure water. Analytical detection was performed using a MARS MOA column (FLM, Guangdong, China) and a refractive index detector with an oven temperature of 50 °C, 5 mM H_2_SO_4_ as the mobile phase, and a flow rate of 0.5 mL/min.

## Supplementary Information

**Additional file 1:** **Table S1.** Strains and plasmids used in this study. **Table S2.** Primers used in this study. **Fig. S1.** Predict the transmembrane domain of MenA in *B. subtilis*. **Fig. S2.** Colony PCR of the engineered strain BS011.**Fig. S3.** Fluorescence intensity curve of amplified pos5P. **Note S1.** Deducing the overall stoichiometry of glycerol conversion to DMK-7 in *B. subtilis*. **Note S2.** Abbreviations. **Note S3.** Supplementary sequence.

## Data Availability

All data generated or analysed during this study are included in this published article and its Additional file 1.

## Enzymes

MenF: Isochorismate synthase
MenD: 2-Succinyl-5-enolpyruvyl6-hydroxy-3-cyclohexene-1-carboxylate synthase
MenE: *O*-Succinylbenzoic acid-CoA ligase
MenB: 1,4-Dihydroxy-2-naphthoyl-CoA synthase
YuxO: 1,4-Dihydroxy-2-naphthoyl-CoA hydrolase
MenA: 1,4-Dihydroxy-2-naphthoate heptaprenyltransferase
MenG: Demethylmenaquinone methyltransferase
IspA: Farnesyl diphosphate synthase
HepS/HepT: Heptaprenyl diphosphate synthase component I/II
DXS: 1-Deoxyxylulose-5-phosphate synthase
DXR: 1-Deoxyxylulose-5-phosphate reductase
IspH: 4-Hydroxy-3-methylbut-2-enyl diphosphate reductase
Fni: Isopentenyl-diphosphate delta-isomerase.
AroA: 3-Deoxy-7-phosphoheptulonate synthase
AroK: Shikimate kinase
AroE: 3-Phosphoshikimate 1-carboxyvinyltransferase
ZWF: Glucose-6-phosphate 1-dehydrogenase
pos5P: NADH kinase
AmyE: Alpha-amylase
HsUBIAD1: Homo sapiens UbiA prenyltransferase containing 1

## Metabolites

Gly: Glycerol
G6P: Glucose-6-phosphate
F6P: Fructose-6phosphate
FBP: Fructose-1,6-bisphosphate
G3P: Glyceraldehyde-3-phosphate
PEP: Phosphoenolpyruvate
PYR: Pyruvate
E4P: Erythrose 4-phosphate
DAHP: 3-Deoxy-arabino-heptulonate 7-phosphate
DHS: 3-Dehydroshikimate
SA: Shikimate
S3P: Shikimate 3-phosphate
CHA: Chorismite
DXP: 1-Deoxyxylulose-5-phosphate
MEP: Methyl-erythritol-4-diphosphate
HMBPP: 1-Hydroxy-2-methyl-2-butenyl 4-diphosphate
DMAPP: Dimethylallyl diphosphate
IPP: Isopentenyl diphosphate
FPP: Farnesyl diphosphate
HepPP: Heptaprenyl diphosphate
ICHA: Isochorismate
SEPHCHC: 2-Succinyl-5-enolpyruvyl-6-hydroxy-3-cyclohexene-1-carboxylate
OSB: 2-Succinylbenzoate
OSB-CoA: 2-Succinyl benzoyl-CoA
DHNA-CoA: 1,4-Dihydroxy-2-naphthoyl-CoA
DHNA: 1,4-Dihydroxy-2-naphthoate
DHNA-CoA: 1,4-Dihydroxy-2-naphthoyl-CoA
DMK-7: Demethylmenaquinone-7
MK-7: Menaquinone-7
AKG: 2-Oxoglutarate
MEP: 2-C-methyl-*d*-erythritol 4-phosphate
