# Supplementary material for: Bottom-up synthetic biology approach for improving the efficiency of menaquinone-7 synthesis in Bacillus subtilis
Source: Microb Cell Fact. 2022 May 28;21:101. doi: 10.1186/s12934-022-01823-3 (PMC9148487; doi:10.1186/s12934-022-01823-3)
Supplement: Supplementary file 1 — Additional file 1: Table S1. Strains and plasmids used in this study. Table S2. Primers used in this study. Fig. S1. Predict the transmembrane domain of MenA in B. subtilis. Fig. S2. Colony PCR of the engineered strain BS011.Fig. S3. Fluorescence intensity curve of amplified pos5P. Note S1. Deducing the overall stoichiometry of glycerol conversion to DMK-7 in B. subtilis. Note S2. Abbreviations. Note S3. Supplementary sequence. [file 12934_2022_1823_MOESM1_ESM.docx]

Additional file 1

Table S1. Strains and plasmids used in this study

Table S2. Primers used in this study

Fig. S1 Predict the transmembrane domain of MenA in *B. subtilis*

Fig. S2 Colony PCR of the engineered strain BS011

Fig. S3 Fluorescence intensity curve of amplified *pos5P*

Note S1. Deducing the overall stoichiometry of glycerol conversion to DMK-7 in *B.subtilis*

Note S2. Abbreviations

Note S3. Supplementary sequence

Table S1. Strains and plasmids used in this study

| Strain | characteristics | reference |
| --- | --- | --- |
| BS168 | trpC2 | Lab stock |
| BS001 | BS168,P_43_-*dxs* | This work |
| BS002 | BS168, P_43_-*dxs*, P_43_-*fni* | This work |
| BS004 | BS168, P_43_-*dxs*, P_43_-*fni*, P_43_-*dxr*, P_43_-*menF* | This work |
| BS005 | BS168, P_43_-*dxs*, P_43_-*fni*, P_43_-*dxr*, P_43_-*menF*, P_hbs_-*aroA* | This work |
| BS006 | BS168, P_43_-*dxs*, P_43_-*fni*, P_43_-*dxr*, P_43_-*menF*, P_hbs_-*aroA*, Δ*dhbB* | This work |
| BS007 | BS168, P_43_-*dxs*, P_43_-*fni*, P_43_-*dxr*, P_43_-*menF*, P_hbs_-*aroA*, Δ*dhbB*::P_sigw_ -*menA* | This work |
| BS008 | BS168, P_43_-*dxs*, P_43_-*fni*, P_43_-*dxr*, P_43_-*menF*, P_hbs_-*aroA*, Δ*dhbB*::P_43_-*menA* | This work |
| BS009 | BS168, P_43_-*dxs*, P_43_-*fni*, P_43_-*dxr*, P_43_-*menF*, P_hbs_-*aroA*, Δ*dhbB*::P_43_-*mstX* -linker-*menA* | This work |
| BS010 | BS168, P_43_-*dxs*, P_43_-*fni*, P_43_-*dxr*, P_43_-*menF*, P_hbs_-*aroA*, Δ*dhbB*::P_43_-*menA*, P_hbs_-*zwf* | This work |
| BS011 | BS168, P_43_-*dxs*, P_43_-*fni*, P_43_-*dxr*, P_43_-*menF*, P_hbs_-*aroA*, Δ*dhbB*::P_43_-*menA*, Δ*amyE*::P_43_-*pos5P* | This work |
| Plasmids |  |  |
| p7S6P43 | pMD18-T ligated with lox71-spc-lox66 cassette and P_43_ promoter | Lab stock |
| p7C6P43 | pMD18-T ligated with lox71-cm-lox66 cassette and P_43_ promoter | Lab stock |
| PDGC | Amp, Km, *E. coli*−*B. subtilis* shuttle vector, containing *cre* under the control of P_spac_ | Lab stock |

Table S2. Primers used in this study

| Primers | Sequences（5＇→3＇） |
| --- | --- |
| dxr- L-F | caggttgcctacagtgaatttgtctttac |
| dxr-L-R | aattgttatccgctcagttgccactactcctattctt |
| dxr-Z-F | aataggagtagtggcaactgagcggataacaatttcac |
| dxr-Z-R | gacaaatatttttcaagtgtacattcctctcttacC |
| dxr-R-F | Ggtaagagaggaatgtacacttgaaaaatatttgtct |
| dxr-R-R | tcggcattgttcctcctattttacctg |
| dxs- L-F | gcttgatatgccggactcattaaag |
| dxs- L-R | gttatccgctcagcggatcaactcactttcagc |
| dxs -Z-F | atgctgaaagtgagttgatccgctgagcggataa |
| dxs -Z-R | gtattgataaaagatccaagtgtacattcctctct |
| dxs -R-F | Ggtaagagaggaatgtacacttggatcttttatcaataca |
| dxs -R-R | gtaccatgccatgtcccaatcgt |
| fni- L-F | taaagtagacgacgagcttgacctgaaag |
| fni- L-R | attgttatccgctcgtttatcaccaattattttagt |
| fni-Z-F | ataattggtgataaacgagcggataacaatttcacacag |
| fni-Z-R | cgttctgctcgagtcacgtgtacattcctctcttacC |
| fni-R-F | gtaagagaggaatgtacacgtgactcgagcagaacg |
| fni-R-R | ccagcacggtcataatcaactttaattcc |
| menF- L-F | tgtggtcatgattccctcttcgtc |
| menF- L-R | cggtacaatctctagagggagacattcctccataatcct |
| menF -Z-F | ggattatggaggaatgtctccctctagagattgtaccgtt |
| menF -Z-R | GCTGCACCGTTGTCACCATGTGTACATTCCTCTCTTACCTAT |
| menF -R-F | GTAAGAGAGGAATGTACACatggtgacaacggtgcagcgtac |
| menF -R-R | GTCGTCTTCTTCATTCACGCCTCGTT |
| aroA-L-F | aaacgacggccagtgaattcCGGTAGCGGCATTAAAATTAG |
| aroA-L-R | atccgctcTTTTTCCACCTCAATCAGCAT |
| aroA-Z-F | GAGGTGGAAAAAGAGCGGATAACAATTTC |
| aroA-Z-R | TCCTTGATCCTTACCGTTCGTATAATGTATGC |
| Phbs-F | CGAACGGTAAGGATCAAGGAATAGGATGAAAAAAG |
| phbs-R | gcaccttatctcgtttcatGTGTACATTCCTCTCTTAC |
| aroA-R-F | cATGAAACGAGATAAGGTGCAGACC |
| aroA-R-R | accatgattacgccaagcttGCCGCGTCCTTAATAACG |
| dhbB-L-F | aaacgacggccagtgaattcTGTACTTGGCCGCGCTGC |
| dhbB-L-R | ctctagagATGAACACTTCCTCTCAAATTGTTGT |
| spc-F | GGAAGTGTTCATCTCTAGAGATTGTACCGTTC |
| spc-R | GTGTACATTCCTCTCTTACCTATAATGG |
| MIS-F | GGTAAGAGAGGAATGTACACATGTTTTGTACATTTTTTG |
| MIS-R2 | tttggttcatCGACCCACCACCGCCCGA |
| menA-F | tggtgggtcgATGAACCAAACAAATAAGGGTGAGG |
| menA-R | GTTTACCCTCCTTTTATCGGAAATAGCTGATC |
| dhbB-R -F | CGATAAAAGGAGGGTAAACAAACGGATG |
| dhbB-R -R | accatgattacgccaagcttATACTTATGATGTCGCCGAATACT |
| MIS-R1 | CGACCCACCACCGCCCGAGCCACCGCCACCttctttttctccttctt |
| Psigw-spc-R2 | Cagtcgacgattctaccgttcgtat |
| Spc-Psigw-F3 | aacggtagaatcgtcgacTGCCCCCCTCCACCATTAT |
| MenA-Psigw-R3 | ttcctctcttacACCGGTCTGTATGTATACGAG |
| Psigw-menA-F4 | AGACCGGTgtaagagaggaatgtac |
| menA-P43-R2 | ggttcatGTGTACATTCCTCTCTTACCTATAATGG |
| P43-menA-F3 | GAGGAATGTACACatgaaccaaacaaataagggtg |
| aym-L-F | gtatgtcaagtggctgcggtttatggt |
| aym-L-R | gtgaaattgttatccgctctcttgacactccttatttg |
| aym-cm-F | caaataaggagtgtcaagagagcggataacaatttcac |
| pos-p43-R | AATGTGAATCCAACGTACTgtgtacattcctctcttacC |
| pos5p-F | GgtaagagaggaatgtacacAGTACGTTGGATTCACATT |
| pos5p-R | tcccgtctagccttgcccTTAATCATTATCAGTCTGTCTCT |
| aym-R-F | AGAGACAGACTGATAATGATTAAgggcaaggctagac |
| aym-R-R | accgtattgcccgtccaaatatgtgct |
| **qRT-PCR** |  |
| dxr-qpcr.F | GCTGGTATCTATGTCGTT |
| dxr-qpcr.R | TATCAGTCCTTCTTCTCC |
| dxs-qpcr.F | AAAAAAGGGAAGGGGTA |
| dxs-qpcr.R | CAGGAAGGTTGAGTAAAT |
| fni-qpcr.F | CGAAAGGAAAACCCAAAC |
| fni-qpcr.R | TCATACCGAAGCCGACT |
| menf-qpcr.F | GATGGGTAAGCGGAGGAGA |
| menf-qpcr.R | GATTGGACCGTCAAACGTG |
| mena-qpcr.F | TGAAGAGGACAAAAAAGGC |
| mena-qpcr.R | GAGCAGGAATCCGAAAAAT |
| aroa-qpcr.F | CTCAGGCGGAAGGAACCAC |
| aroa-qpcr.R | CAGGAAGCAATACCAAGCA |
| dhbb-qpcr.F | GAGGATGATGATCTTGTGC |
| dhbb-qpcr.R | TTCTTTTTGCCAGTGTTTG |
| Pos5p-qpcr.F | TGAAAAAAAAGGATAGCAAC |
| Pos5p-qpcr.R | GACAAAGAGCGAGGACAA |
| hbs-qpcr.F | AACAGAACTTATCAATGCGGT |
| hbs-qpcr.R | AGGTACTTTGCTTGCTGGAAT |


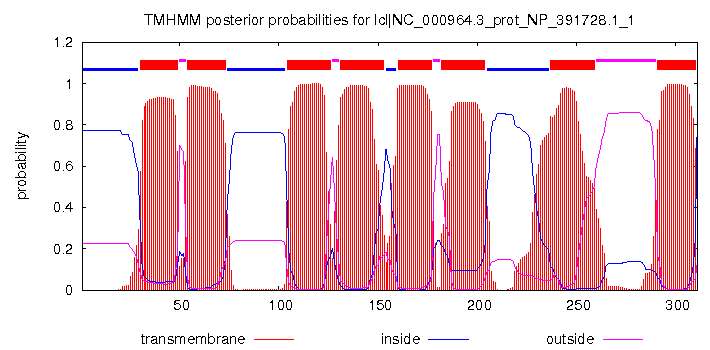


Fig. S1 Predict the transmembrane domain of MenA in *B. subtilis*. There are eight transmembrane helices in the membrane protein MenA.


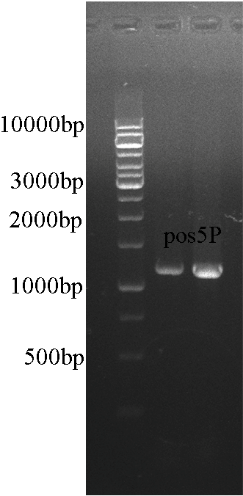


Fig. S2 Colony PCR of the engineered strain BS011. Lane 1, DL 10kp DNA Marker; Lane 2 and 3, the truncated *pos5p*


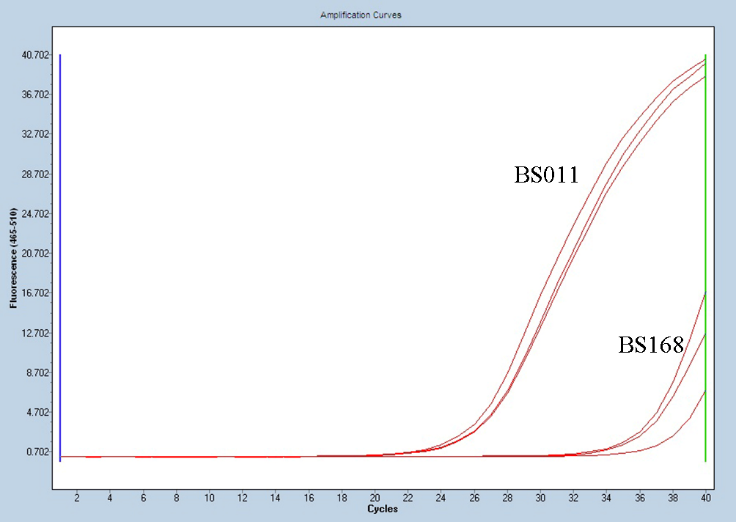


Fig. S3 Fluorescence intensity curve of amplified *pos5p*. The mean Ct of the *pos5p* in BS011 was 18.57, while the mean Ct of that in BS168 was so small that it can be ignored.

Note S1. Deducing the overall stoichiometry of glycerol (Gly) conversion to MK-7 in *B. subtilis*

In *B. subtilis*, MK-7 biosynthetic pathways were shown in Fig. 1 (described in the paper), the process requires five precursors (glyceraldehyde-3-phosphate, phosphoenolpyruvate, pyruvate, erythrose 4-phosphate, 2 -oxoglutarate). The overall pathway from Gly to MK-7 was recast into eight parts (all reactions are based on the Kyoto Encyclopedia of Genes and Genomes): synthesis of precursors glyceraldehyde-3-phosphate(G3P), phosphoenolpyruvate(PEP), pyruvate(PYR), erythrose 4-phosphate(E4P), 2-oxoglutarate(AKG), 1,4-dihydroxy2-naphthoate(DHNA), heptaprenyl diphosphate(HepPP) and synthesis of demethylmenaquinone-7(DMK-7). For calculation purposes, we assumed that all carbon could be converted to DMK-7, and the elemental balances for oxygen and hydrogen were not considered.

Part 1: Synthesis of G3P, PEP and PYR. The uptake of Gly is catalyzed in an energy-independent manner by a membrane channel protein, the glycerol facilitator GlpF. The main pathway of glycerol dissimilation involves a glycerol kinase GlpK that phosphorylates Gly to glycerol-3-phosphate (Gly-3P), and a Gly-3P dehydrogenase GlpD that oxidizes Gly-3P to dihydroxyacetone phosphate (DHAP), an intermediate in glycolysis. The reactions of glycerol phosphorylation and conversion to G3P are shown as follows:

$$Gly+ATP\longrightarrow{Gly­3+ADP}$$

$$Gly­3{+FAD}^{+}\longrightarrow{DHAP+\mathrm{FADH}_{2}}$$

$$\mathrm{DHAP}\longrightarrow{G3P}$$

$$G3P{+NAD}^{+}\longrightarrow\mathrm{BPG}{+NADH}$$

$${BPG+ADP}\longrightarrow3PG{+ATP}$$

$${3PG}\longrightarrow2PG$$

$${2PG}\longrightarrow\mathrm{PEP}$$

$${PEP+ADP}\longrightarrow PYR+ATP$$

The overall reaction of G3P synthesis from Gly is:

1. ${Gly+ATP+FAD}^{+}\longrightarrow{G3P+ADP+FADH}_{2}$

The overall reaction of PEP synthesis from Gly is:

1. ${Gly+NAD}^{+}{+FAD}^{+}\longrightarrow PEP+NADH+\mathrm{FADH}_{2}$

The overall reaction of PYR synthesis from Gly is:

1. ${Gly+NAD}^{+}{+FAD}^{+}+ADP\longrightarrow PYR+NADH+\mathrm{FADH}_{2}+ATP$

Part 2: Synthesis of E4P. DHAP is catalyzed by fructose bisphosphate aldolase (FbaA) to produce fructose-1, 6-bisphosphate (FBP). Glucose 6-phosphate (G6P) is then generated through a series of catalysis, which enters the pentose phosphate pathway under the catalysis of glucose-6-phosphate 1-dehydrogenase (ZWF). E4P is finally generated, and the reaction equations are as follows:

$$2Gly+{2FAD}^{+}+ATP\longrightarrow2DHAP+{2FADH}_{2}+2ADP$$

$${2DHAP}\longrightarrow\mathrm{FBP}$$

$$\mathrm{FBP}\longrightarrow F6P$$

$${F6P}\longrightarrow G6P$$

$$G6P{+NADP}^{+}\longrightarrow6PGNL+NADPH$$

$$6PGNL\longrightarrow6PG$$

$$6PG{+NADP}^{+}\longrightarrow R_{u}5P+NADPH+\mathrm{CO}_{2}\uparrow$$

$${2R}_{u}5P\longrightarrow E4P+F6P$$

$$2\mathrm{Gly}{+2NADP}^{+}+{2FAD}^{+}+ATP\longrightarrow R_{u}5P+2NADPH+{2FADH}_{2}+2ADP+phosphate+\mathrm{CO}_{2}\uparrow$$

$$4\mathrm{Gly}{+4NADP}^{+}+{4FAD}^{+}+4ATP\longrightarrow E4P+F6P+4NADPH+{4FADH}_{2}+4ADP+{2CO}_{2}\uparrow$$

$$2Gly+{2FAD}^{+}+2ATP\longrightarrow F6P+{2FADH}_{2}+2ADP$$

The overall reaction of PYR synthesis from Gly is:

1. $2\mathrm{Gly}{+4NADP}^{+}+{2FAD}^{+}+2ATP\longrightarrow E4P+4NADPH+{2FADH}_{2}+2ADP+{2CO}_{2}\uparrow$

Part 3: Synthesis of AKG. AKG is an intermediate in the TCA cycle. First, PYR is catalyzed by the pyruvate dehydrogenase complex to generate acetyl-COA and enter the TCA cycle. Then it is catalyzed by citrate synthase (CitA), aconitate hydratase (CitB), and isocitron dehydrogenase (Icd) in turn to generate AKG. The reaction equations are as follows:

$$\mathrm{PYP}{+NAD}^{+}\longrightarrow AcCoA+NADH+\mathrm{CO}_{2}\uparrow$$

$$AcCoA+OAA\longrightarrow\mathrm{Cit}$$

$$\mathrm{Cit}\longrightarrow\mathrm{Icit}$$

$$\mathrm{Icit}{+4NADP}^{+}\longrightarrow AKG+NADPH+\mathrm{CO}_{2}\uparrow$$

$$\mathrm{Gly}{+OAA+NADP}^{+}{+2NAD}^{+}+\mathrm{FAD}^{+}+ADP\longrightarrow AKG+2NADH+NADPH+\mathrm{FADH}_{2}+ATP+{2CO}_{2}\uparrow$$

$$OAA+ATP\longrightarrow PYR+ADP+{2CO}_{2}\uparrow$$

$$\mathrm{Gly}{+PYR+NADP}^{+}{+2NAD}^{+}+\mathrm{FAD}^{+}+2ADP\longrightarrow AKG+2NADH+NADPH+\mathrm{FADH}_{2}+2ATP+\mathrm{CO}_{2}\uparrow$$

The overall reaction of AKG synthesis from Gly is:

1. ${2Gly+{3NAD}^{+}+NADP}^{+}{+2FAD}^{+}+3ADP\longrightarrow AKG+NADPH+3NADH+{2FADH}_{2}+2ADP+\mathrm{CO}_{2}\uparrow$

Part 4: Synthesis of DHNA. Chorismate (CHA) is synthesized via the shikimate pathway from two precursors: E4P and PEP. Then isochorismate synthase (MenF) converts CHA into isochorismate(ICHA), which is further converted by six enzymes encoded by the *menFDHBEC* operon to form DHNA. The reaction equations are as follows:

$$E4P+PEP\longrightarrow DHAP$$

$$DHAP\longrightarrow DHQ$$

$$DHQ\longrightarrow DHS$$

$$DHS+NADPH\longrightarrow SA+\mathrm{NADP}^{+}$$

$$SA+ATP\longrightarrow S3P+ADP$$

$$S3P+PEP\longrightarrow EPSP$$

$$EPSP\longrightarrow CHA$$

$$CHA\longrightarrow ICHA$$

$$AKG+ICHA\longrightarrow SEPHCHC+\mathrm{CO}_{2}\uparrow$$

$$\mathrm{SEPHCHC}\longrightarrow SHCHC+PYR$$

$$\mathrm{SHCHC}\longrightarrow\mathrm{OSB}$$

$$OSB+ATP+COA\longrightarrow OSB­COA+AMP$$

$$OSB­COA+H^{+}\longrightarrow DHNA­COA$$

$$DHNA­COA\longrightarrow DHNA+COA$$

$$AKG+E4P+2PEP+NADPH+2ATP\longrightarrow DHNA+PYR+ADP+\mathrm{NADP}^{+}+AMP+\mathrm{CO}_{2}\uparrow$$

The overall reaction of DHNA synthesis from Gly is:

1. ${5Gly+{4NAD}^{+}+4NADP}^{+}{+5FAD}^{+}+2ATP\longrightarrow DHNA+4NADPH+4NADH+{5FADH}_{2}+ADP+AMP+{4CO}_{2}\uparrow$

Part 5: Synthesis of HepPP. PYR will also condense with G3P to form 1-deoxy-D-xylose-5-phosphate (DXP) and enter the methyl-erythritol-4-diphosphate (MEP) pathway for isopentenyl diphosphate (IPP) formation. And then IPP and its isomer dimethylallyl diphosphate (DMAPP) are catalyzed by farnesyl diphosphate synthase (FPPS) and heptaprenyl diphosphate synthase component I/II (HepS/HepT) to generate HepPP. The reaction equations are as follows:

$$G3P+PYR\longrightarrow DXP+\mathrm{CO}_{2}\uparrow$$

$$DXP+NADPH\longrightarrow MEP+\mathrm{NADP}^{+}$$

$$MEP+CTP\longrightarrow CDP­ME$$

$$CDP­ME+ATP\longrightarrow CDP­MEP+ADP$$

$$CDP­MEP\longrightarrow MEC+CMP$$

$$\mathrm{MEC}\longrightarrow\mathrm{HMBPP}$$

$$HMBPP+NADPH\longrightarrow IPP+\mathrm{NADP}^{+}$$

$$HMBPP+NADPH+H^{+}\longrightarrow DMAPP+\mathrm{NADP}^{+}$$

$$G3P+PYR+2NADPH+CTP+ATP\longrightarrow IPP+ADP+CMP+{2NADP}^{+}+\mathrm{CO}_{2}\uparrow$$

$$2Gly+2NADPH+2\mathrm{FAD}^{+}{+NAD}^{+}+CTP+ATP\longrightarrow IPP+NADH+2\mathrm{FADH}_{2}+ADP+CMP+{2NADP}^{+}+\mathrm{CO}_{2}\uparrow$$

The overall reaction of HepPP synthesis from Gly is:

1. $14Gly+14NADPH+14\mathrm{FAD}^{+}{+7NAD}^{+}+7CTP+7ATP\longrightarrow HepPP+7NADH+14\mathrm{FADH}_{2}+7ADP+7CMP+{14NADP}^{+}+7\mathrm{CO}_{2}\uparrow$

Part 6: Synthesis of DMK-7. The polyisoprene tail is ligated with the naphthoquinone head by MenA to form DMK-7 and MK-7 is synthesized via the methylation of DMK-7. The reaction equations are as follows:

$$DHNA+HepPP\longrightarrow DMK­7+\mathrm{CO}_{2}\uparrow$$

The overall reaction of DMK-7 synthesis from Gly is:

1. $19Gly+{11NAD}^{+}+10NADPH{+19FAD}^{+}+7CTP+9ATP\longrightarrow DMK­7+{10NADP}^{+}+11NADH+{19FADH}_{2}+8ADP+AMP+7CMP+{12CO}_{2}\uparrow$

Note S2. Abbreviations

Gly, Glycerol; Gly­3, glycerol-3-phosphate; DHAP, dihydroxyacetone phosphate; $\mathrm{BPG}$, Glyceroyl-1,3P_2_, 3PG, Glycerate-3P; 2PG, Glycerate-2P; G6P, glucose-6-phosphate; F6P, fructose-6phosphate; FBP, fructose-1,6-bisphosphate;G3P, glyceraldehyde-3-phosphate; PEP, phosphoenolpyruvate; PYR, pyruvate; E4P, erythrose 4-phosphate; DAHP, 3-deoxy-arabino-heptulonate 7-phosphate; 6PGNL, 6-phospho-D-glucono-1,5-lactone;6PG, 6-Phospho-D-gluconate; Ru5P, D-Ribulose 5-phosphate; AcCoA, Acetyl-CoA; OAA, Oxaloacetate; cit, citrate; Icit, Isocitrate; DHQ, 3-Dehydroquinate; DHS, 3-dehydroshikimate; SA, shikimate; S3P, shikimate 3-phosphate; CHA, Chorismite; DXP, 1-deoxyxylulose-5-phosphate; MEP, methyl-erythritol-4-diphosphate; HMBPP, 1-hydroxy-2-methyl-2-butenyl 4-diphosphate; DMAPP, dimethylallyl diphosphate; IPP, isopentenyl diphosphate; FPP, farnesyl diphosphate; HepPP, heptaprenyl diphosphate; ICHA, isochorismate; SEPHCHC, 2-succinyl-5-enolpyruvyl-6-hydroxy-3-cyclohexene-1-carboxylate; OSB, 2-succinylbenzoate; OSB-CoA, 2-succinyl benzoyl-CoA; DHNA-CoA, 1,4-dihydroxy-2-naphthoyl-CoA; DHNA, 1,4-dihydroxy2-naphthoate; DHNA-CoA, 1,4-Dihydroxy-2-naphthoyl-CoA; DMK-7, demethylmenaquinone-7; MK-7, menaquinone-7；AKG, 2-oxoglutarate; MEP: 2-C-methyl-d-erythritol 4-phosphate;

Note S3. Supplementary sequence

P_hbs_

AGGATCAAGGAATAGGATGAAAAAAGGAAAAAAAGGAATATTCGTTCGGTAAATCACCTTAAATCCTTGACGAGCAAGGGATTGACGCTTTAAAATGCTTGATATGGCTTTTTATATGTGTTACTCTACATACAGAAATTCTTCACTTTGTTGGACAAACATTCCTCAGAGTGCAGTTTTTCTTAAAAAGCCGTTTAATTGTCTTTCTCTTACTTGCTCTCATTTTTTTCTGAGACAGGTTTAGAATCAGACTGAACTGTGAAGAAATGATAATAAACGAACTGAATGTATCCTTTTGGG

*mstX*

Atgttttgtacattttttgaaaaacatcaccggaagtgggacatactgttagaaaaaagcacgggtgtgatggaagctatgaaagtgacgagtgaggaaaaggaacagctgagcacagcaatcgaccgaatgaatgaaggactggacgcgtttatccagctgtataatgaatcggaaattgatgaaccgcttattcagcttgatgatgatacagccgagttaatgaagcaggcccgagatatgtacggccaggaaaagctaaatgagaaattaaatacaattattaaacagattttatccatctcagtatctgaagaaggagaaaaagaa

Linker

ACTAGTGGTGGTGGTGGTTCTGGTGGTGTGGATCCGGTGGCGTGGTTCTGCATGC

P_sigw_

TGCCCCCCTCCACCATTATTGGGCTATAGCCAAGCGGTAAGGCAACGGACTTTGACTCCGTCATGCGTTGGTTCGAATCCAGCTAGCCCAGTCACAGACACCTTTGATCAAAAGGTGTCTTTTTTCTTTTCGGAAAAATCATTCCAACTTCTAACTGTTCAGTCTGTATAATAATTTTAAAAATATGTTAAGGTAGTTTATTCACGAATTACCATCTACACCCTGCCAAAAATTTGATAAACTTATTTTATAAAAAAATTGAAACCTTTTGAAACGAAGCTCGTATACATACAGACCGGT

The truncated *pos5P*

AGTACGTTGGATTCACATTCCCTAAAGTTACAGAGCGGCTCGAAGTTTGTAAAAATAAAGCCAGTAAATAACTTGAGGAGTAGTTCATCAGCAGATTTCGTGTCCCCACCAAATTCCAAATTACAATCTTTAATCTGGCAGAACCCTTTACAAAATGTTTATATAACTAAAAAACCATGGACTCCATCCACAAGAGAAGCGATGGTTGAATTCATAACTCATTTACATGAGTCATACCCCGAGGTGAACGTCATTGTTCAACCCGATGTGGCAGAAGAAATTTCCCAGGATTTCAAATCTCCTTTGGAGAATGATCCCAACCGACCTCATATACTTTATACTGGTCCTGAACAAGATATCGTAAACAGAACAGACTTATTGGTGACATTGGGAGGTGATGGGACTATTTTACACGGCGTATCAATGTTCGGAAATACGCAAGTTCCTCCGGTTTTAGCATTTGCTCTGGGCACTCTGGGCTTTCTATCACCGTTTGATTTTAAGGAGCATAAAAAGGTCTTTCAGGAAGTAATCAGCTCTAGAGCCAAATGTTTGCATAGAACACGGCTAGAATGTCATTTGAAAAAAAAGGATAGCAACTCATCTATTGTGACCCATGCTATGAATGACATATTCTTACATAGGGGTAATTCCCCTCATCTCACTAACCTGGACATTTTCATTGATGGGGAATTTTTGACAAGAACGACAGCAGATGGTGTTGCATTGGCCACTCCAACGGGTTCCACAGCATATTCATTATCAGCAGGTGGATCTATTGTTTCCCCATTAGTCCCTGCTATTTTAATGACACCAATTTGTCCTCGCTCTTTGTCATTCCGACCACTGATTTTGCCTCATTCATCCCACATTAGGATAAAGATAGGTTCCAAATTGAACCAAAAACCAGTCAACAGTGTGGTAAAACTTTCTGTTGATGGTATTCCTCAACAGGATTTAGATGTTGGTGATGAAATTTATGTTATAAATGAGGTCGGCACTATATACATAGATGGTACTCAGCTTCCGACGACAAGAAAAACTGAAAATGACTTTAATAATTCAAAAAAGCCTAAAAGGTCAGGGATTTATTGTGTCGCCAAGACCGAGAATGACTGGATTAGAGGAATCAATGAACTTTTAGGATTCAATTCTAGCTTTAGGCTGACCAAGAGACAGACTGATAATGAT
